# A Case of Rapidly Progressive De Novo Metastatic Small-Cell Neuroendocrine Prostate Cancer

**DOI:** 10.1155/2024/7998149

**Published:** 2024-09-17

**Authors:** Aryan Dalal, Sean Clark-Garvey, Andrew Gdowski, Sophia Zhang, Sara E. Wobker, Steven P. Rowe, Ersan Altun, Himisha Beltran, Matthew I. Milowsky

**Affiliations:** ^1^ Department of Biostatistics University of North Carolina, Chapel Hill, North Carolina, USA; ^2^ Lineberger Comprehensive Cancer Center University of North Carolina, Chapel Hill, North Carolina, USA; ^3^ Department of Medicine University of North Carolina School of Medicine, Chapel Hill, North Carolina, USA; ^4^ Department of Pathology and Laboratory Medicine University of North Carolina School of Medicine, Chapel Hill, North Carolina, USA; ^5^ Department of Radiology University of North Carolina School of Medicine, Chapel Hill, North Carolina, USA; ^6^ Department of Medical Oncology Dana-Farber Cancer Institute Harvard Medical School, Boston, Massachusetts, USA

**Keywords:** case report, prostate cancer, small-cell/neuroendocrine carcinoma, treatment of neuroendocrine prostate cancer

## Abstract

**Introduction:** Neuroendocrine/small-cell prostate cancer (NEPC) is a rare and aggressive subtype of prostate cancer, which typically develops after prolonged treatment for metastatic castration-resistant disease, but can, less commonly, occur de novo.

**Case Presentation:** We describe a case of de novo NEPC in a tumor with mixed pathology including acinar adenocarcinoma and neuroendocrine/small-cell carcinoma with rapid progression of metastatic disease. Despite initiation of treatment with androgen deprivation therapy (ADT) and chemotherapy, the patient continued to exhibit progression leading to multiple complications including a large bowel obstruction and ultimately progressive hepatic metastases resulting in liver failure.

**Conclusion:** This case illustrates the clinical presentation and highly aggressive nature of de novo NEPC. Recognizing atypical clinical progression in prostate cancer is critical for the detection of NEPC; however, despite early identification and initiation of treatment, the prognosis remains poor, thus highlighting the need for further study into NEPC biology and novel therapeutic approaches.

## 1. Introduction

Prostate cancer is the most common malignancy in men in the United States and remains a significant cause of morbidity and mortality [1]. Like many other malignancies, prostate cancer is a heterogeneous disease, although the vast majority of cases are androgen-driven acinar adenocarcinomas [2]. The backbone of systemic therapy for advanced/metastatic prostate adenocarcinoma is androgen deprivation therapy (ADT) administered in conjunction with an androgen receptor pathway inhibitor (ARPI), with or without chemotherapy, in the first-line setting [3]. Despite the established efficacy of such regimens, most patients will progress to and develop metastatic castration-resistant prostate cancer (mCRPC) [4]. In spite of such resistance, most cases of mCRPC remain dependent on androgen signaling; however, a subset (~15%–20%) of patients develop androgen signaling–independent disease [5]. An even smaller subset of patients with androgen-independent disease will develop histologic transformation to an aggressive neuroendocrine/small-cell prostate cancer (NEPC) [5].

The classification of prostate cancer with neuroendocrine differentiation continues to evolve but encompasses tumors with mixed (neuroendocrine/small-cell and adenocarcinoma features) and pure small-cell carcinoma histology [6]. NEPC typically develops in the context of prolonged treatment for metastatic disease, termed treatment-related neuroendocrine/small-cell prostate cancer (t-NEPC). The incidence rate of t-NEPC in patients with mCRPC varies in the literature from 6% to 20% but is likely increasing, possibly secondary to increased utilization of ARSIs along with increased awareness [5, 7]. A rarer entity, with an incidence rate of < 2%, is de novo NEPC, which can also present as a mixed or pure small-cell carcinoma [5, 7]. Both t-NEPC and de novo NEPC are more aggressive when compared to prostatic adenocarcinoma and are associated with a poorer prognosis [8]. Additionally, NEPCs typically have a different phenotypic presentation and are clinically characterized by more frequent visceral metastases, lytic bone lesions, low or normal prostate-specific antigen (PSA) values, and lack of response to androgen inhibition [7].

Here, we report a case of a particularly aggressive de novo metastatic mixed NEPC, which highlights many of the unique clinical features of NEPC and the importance of keeping such a diagnosis in the differential.

## 2. Case Presentation

**Figure 1:**
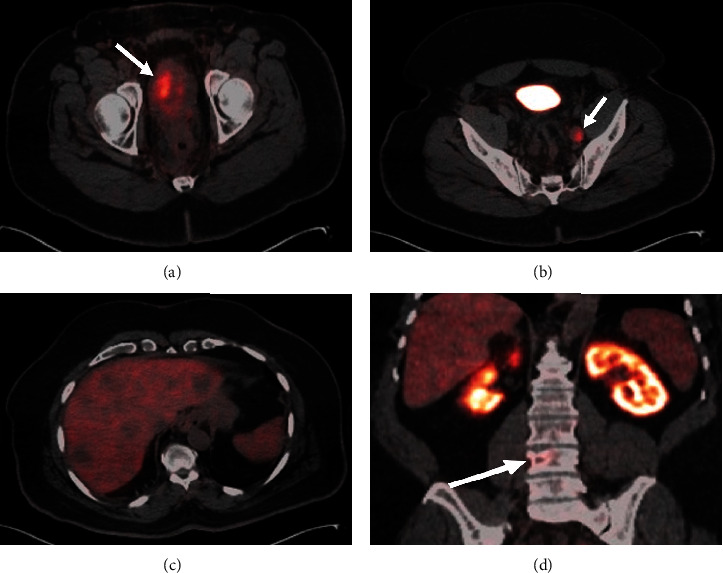
Piflufolastat F-18 PET/CT: (a–c) axial and (d) coronal images showing heterogeneous avidity of radiotracer in the enlarged prostate gland (arrow, a), enlarged pelvic sidewall lymph node with radiotracer avidity (arrow, b), numerous liver lesions showing no or minimal radiotracer avidity (c), and a lytic lesion at the right side of the L4 vertebral body with radiotracer avidity (arrow, d). The findings represent heterogeneous uptake/PSMA expression across the primary tumor and various sites of metastatic disease, consistent with the mixed histology of this patient's disease.

A 64-year-old man with a past medical history of chronic hepatitis B, hypertension, and chronic lower back pain presented with an approximate 1-year history of increased urinary frequency and worsening lower back pain. A PSA level was elevated at 58.44 ng/mL (Table 1), and a digital rectal exam was notable for a firm prostate nodule. A transrectal ultrasound-guided prostate biopsy revealed adenocarcinoma, Gleason Grade Group 5 in 12 of 12 cores, with perineural invasion and cribriform morphology. A pelvic magnetic resonance image (MRI) was notable for a Prostate Imaging Reporting & Data System (PI-RADS) 5 lesion involving the majority of the prostate, extracapsular extension involving the left neurovascular bundle, possible bilateral seminal vesicle involvement, concern for invasion of the anterior rectal wall, multifocal pelvic adenopathy with the largest node measuring 2.1 cm, and osseous lesions involving the pelvis and lumbar spine. A bone scan showed no uptake that would be consistent with metastatic disease. A prostate-specific membrane antigen positron emission tomography (PSMA-PET) scan was obtained with uptake in the prostate (lean body mass–corrected standardized uptake value [SUVmax]: 13.7), multiple avid retroperitoneal and pelvic lymph nodes (SUVmax range: 4.3–8.4), and lytic foci within the left superior pubic ramus and L4 vertebral body (SUVmax range: 4.2–6.5) (Figure 1). Additionally, there were multiple solid lesions in the liver that did not have visible focal uptake (SUVmax up to 1.9).

**Figure 2:**
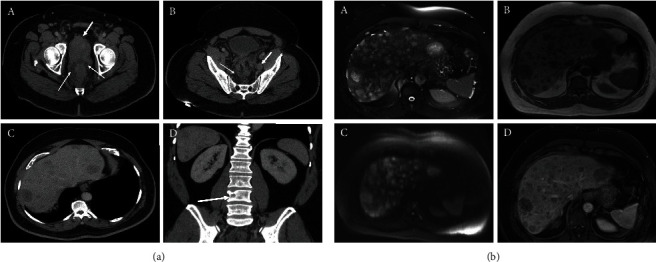
(a) CT images: postcontrast (A–C) axial and (D) coronal images showing the significantly enlarged heterogeneous prostate gland with irregular borders (thick arrow, A) and significant stranding involving the mesorectum (thin arrows, A) with associated rectal wall thickening. Presacral soft tissue involvement characterized as significant stranding is also noted (thin arrow, B) with associated left pelvic side wall enlarged lymph node (thick arrow, B). The findings are suggestive of infiltrative prostate cancer involving extraprostatic adjacent tissues with local extension with metastatic adenopathy. (C) Numerous hypoattenuating liver lesions likely represent bilobar hepatic metastatic disease. A lytic metastatic bone lesion with associated mild pathologic fracture is also noted at the right side of the L4 vertebral body (arrow, D). (b) MRI images: (A) transverse T2-weighted fat-suppressed single-shot echo train spin echo, (B) T1-weighted in-phase spoiled gradient echo, (C) diffusion-weighted, and (D) postgadolinium hepatic venous phase T1-weighted fat-suppressed three-dimensional gradient echo images show numerous bilobar metastatic liver lesions with high T2 signal, low T1 signal, high diffusion signal, and postcontrast rim type of enhancement with associated mild internal hypoenhancement compared to the background liver.

Given the above findings, the patient's initial presentation was consistent with de novo metastatic castration-sensitive prostatic adenocarcinoma. He was initiated on ADT with leuprolide acetate. The plan was to also initiate therapy with a novel ARPI, but this was delayed secondary to poorly controlled hypertension. Within 1–2 weeks of initiating ADT, the patient developed worsening lower back pain and constipation prompting emergency department evaluation. Computed tomography (CT) imaging of the abdomen and pelvis was notable for worsening pelvic adenopathy and lytic metastases, a possible L4 compression fracture, and innumerable liver lesions (Figure 2(a)). His back pain improved with supportive care. He returned to the clinic the following week and was found to have newly elevated liver function enzymes (alanine transaminase [ALT]: 91 U/L; aspartate transaminase [AST]: 64 U/L).

**Figure 3:**
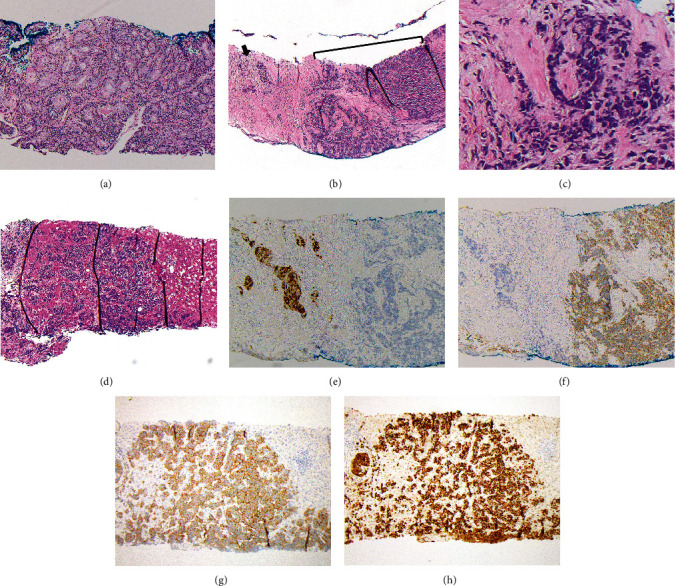
Liver and prostate biopsy, H&E, and immunohistochemistry: prostate core biopsy (a, b) shows conventional prostatic acinar adenocarcinoma with areas of poorly formed glands and infiltrative single cells (arrow) immediately adjacent to small-cell carcinoma in sheets and cords (bracket). On higher power (c), the small-cell carcinoma component demonstrates crush artifact, high nuclear to cytoplasmic ratios, and nuclear molding. Liver lesions (d) biopsied 2 months after the prostate biopsy shows nests of small-cell carcinoma invading through liver parenchyma. In the prostate biopsy (e), the nuclei of infiltrating acinar cell adenocarcinoma stains strongly positive for NKX3.1, a sensitive and specific prostate marker, and the adjacent small-cell carcinoma (f) has strong cytoplasmic granular staining for synaptophysin, a sensitive marker for neuroendocrine differentiation. The liver lesions (g) also stain positive for synaptophysin and strongly positive for INSM1 (h), a highly sensitive and specific neuroendocrine marker, consistent with metastasis of the prostate cancer with neuroendocrine differentiation and small-cell features.

With the findings of elevated ALT and AST and imaging with multiple hepatic lesions, the patient was admitted to expedite workup with concern for neuroendocrine differentiation of prostate cancer. Notably, PSA had declined to 2.62 ng/mL on hormonal therapy (Table 1). An MRI of the abdomen confirmed innumerable hepatic lesions and demonstrated extensive osseous metastatic disease (Figure 2(b)). A liver biopsy was obtained and pathology revealed metastatic disease most consistent with small-cell carcinoma with immunohistochemistry that was diffusely positive for INSM1, synaptophysin, MOC-31, and TTF-1 and focally positive for chromogranin, and Ki-67 labeling index was > 90%; staining was negative for PSA, NKX3.1, CDX2, PAX8, and GATA-3 (Figure 3). In this context, the patient's initial prostate biopsy was re-reviewed, with additional immunostains, and found to show both conventional high-grade acinar prostate adenocarcinoma as well as a component of small-cell carcinoma (Figure 3). The hospitalization was complicated by the development of a large bowel obstruction secondary to prostatic mass invasion of the rectum ultimately requiring palliative surgical intervention with a diverting ostomy. On Postoperative Day 3, following a multidisciplinary discussion with the patient reviewing the risks and benefits of initiating chemotherapy, the decision was made to start carboplatin and etoposide. He also received a short course of palliative radiation to the L3–L5 vertebrae.

The patient initially tolerated chemotherapy well and was discharged in stable condition. Unfortunately, soon after discharge, he was found to be down in an obtunded state at home after a presumed fall. He was readmitted, and CT imaging showed a C5 pars interarticularis fracture and compression fractures of T12 and L4. A brain MRI was negative for metastatic disease. His T12 and L4 fractures were treated with kyphoplasty, and he was again discharged home in stable condition.

The patient was seen in the clinic shortly after discharge, for Cycle 2 of carboplatin/etoposide, but was found to be in severe pain with altered mental status. His labs were notable for worsening liver function and a new acute kidney injury (Table 1). He was readmitted, and a CT of the abdomen/pelvis showed progression of his hepatic metastases and lymphadenopathy. While admitted, his mental status remained altered, liver and kidney function continued to worsen (Table 1), and there was concern for a recurrent bowel obstruction. In that setting, the decision was made to transition to comfort care. The patient passed away shortly thereafter, approximately 4 months after the initial diagnosis of prostate cancer.

## 3. Discussion

The transformation process from prostate adenocarcinoma to NEPC remains to be fully elucidated. Genomic drivers such as loss of function in the tumor suppressor genes *RB1* and *TP53* as well as epigenomic alterations (e.g., changes in DNA methylation and chromatin accessibility) and dysregulation of lineage-associated transcription factors (e.g., ASCL1 and BRN2) have been associated with NEPC and likely contribute to its aggressive phenotype [5]. In the case of t-NEPC, it has been suggested that neuroendocrine differentiation develops as a mechanism of resistance to androgen-targeted therapy, evolving from a luminal/adenocarcinoma cell of origin, and involves lineage reprograming favoring the selection of clones with neuroendocrine features which lack dependence on androgen signaling [5, 9]. De novo NEPCs are by definition not associated with prior treatment and far less are known about their nature, given their rarity. However, de novo disease has been found to harbor some of the same genetic alterations as seen in t-NEPC and prostate adenocarcinoma, suggesting a similar luminal cell of origin of de novo NEPC. In a small study of 18 patients with de novo disease (pure and mixed small-cell carcinoma histology), there was a similar frequency of alterations/deletions of *RB1*, *TP53*, and *PTEN* to t-NEPC. Additionally, 29% of patients were found to have biallelic loss of DNA repair genes (including *BRCA 1/2*, *ATM*, and *MSH2/6*) [10]. The prostate cancer–specific *TMPRSS2-ERG* gene fusion has also been seen in approximately 50% of cases, similar to the rate in adenocarcinoma, further supporting a luminal prostate cell of origin [11]. Unfortunately, we were unable to obtain somatic or germline sequencing on this patient, initially due to the patient's lack of health insurance, and subsequently, the focus of care quickly shifted toward management of his rapid progression and multiple complications.

This patient's clinical course is consistent with a diagnosis of de novo mixed NEPC given the features of both small-cell and high-grade adenocarcinoma seen on his pathology review. Notably, as detailed above, the patient's prostate biopsy was initially felt to be consistent with a high-grade adenocarcinoma, concordant with his significantly elevated PSA. Further, disease in his prostate, retroperitoneal/pelvic lymphadenopathy, and bone was positive on PSMA-PET imaging. He did have a focus of less common lytic bone disease, but it was avid on PSMA imaging. However, the solid, but PSMA-negative, lesions in the liver, as well as the overall heterogeneity of uptake (as shown by a wide range in SUVmax across his lesions), should raise suspicion for NEPC arising out of prostate adenocarcinoma [12]. In carefully selected patients, the interpreting radiologist or nuclear medicine physician should have a low threshold for better characterization of such lesions with liver MRI.

His PSA also declined during therapy, indicating that the adenocarcinoma component was being appropriately suppressed with ADT, but his small-cell carcinoma component was rapidly progressing. Ultimately, his liver biopsy, which was consistent with pure small-cell carcinoma, confirmed the diagnosis. Given the evidence supporting similar genetic alterations in NEPC and prostate adenocarcinoma, we speculate that our patient's mixed histology may have developed from a similar cell of origin through divergent differentiation with the progression of the small-cell carcinoma clone leading to his phenotypic presentation. This would also be consistent with the lineage reprograming thought to drive t-NEPC in patients with a history of CRPC.

The current standard front-line treatment for small-cell NEPC involves either cytotoxic platinum-based regimens alone or in combination with anti-PD-L1 checkpoint inhibitors, regimens largely extrapolated from those used for small-cell lung cancer [6, 13, 14]. Carboplatin plus etoposide is a commonly used regimen in the front-line setting, with carboplatin plus a taxane (docetaxel or cabazitaxel) being a common alternative regimen [13]. The established efficacy of both docetaxel and cabazitaxel in metastatic CRPC provided a rationale for the selection of a taxane-based regimen in the setting of NEPCs with mixed histology; however, in the context of the patient's worsening liver function, including a Grade 3 transaminitis, the regimen of carboplatin/etoposide was chosen. Concomitant therapy with hormonal agents (i.e., ADT and ARPI) may also be indicated in patients with mixed histology to treat the component of adenocarcinoma which may remain dependent on androgen signaling, as was the case in our patient.

NEPCs are typically sensitive to cytotoxic chemotherapy, but the responses are short-lived. Our patient was only able to receive one cycle of carboplatin/etoposide and did demonstrate signs of clinical response with improvement in his liver function (Table 1). Unfortunately, his second cycle was delayed due to his admission for vertebral compression fractures, and he then developed rapid disease progression with marked worsening liver function and renal failure (Table 1). That rapid progression over the course of weeks highlights the particularly aggressive nature of de novo NEPC.

Small-cell NEPCs are aggressive tumors and are associated with a poor prognosis. In one study of 87 patients with histologically confirmed NEPC (both de novo NEPC and t-NEPC), the median overall survival for de novo NEPC was 16.8 months [8]. Notably, this patient passed away 4 months after his initial diagnosis. The shorter survival is likely multifactorial, but given the patient's extensive and rapidly progressive metastatic disease, we suspect he had a particularly aggressive variant.

## 4. Conclusion

This case highlights many of the unique clinical features of small-cell NEPC. It demonstrates the complexity that can occur when a patient presents with clinical and pathologic features consistent with the common diagnosis of prostatic adenocarcinoma. This case also demonstrates the highly aggressive nature of small-cell NEPC and the importance of keeping NEPC in the differential diagnosis, particularly if a patient's clinical course is inconsistent with that of a more typical indolent adenocarcinoma. Despite early recognition of NE differentiation, it remains a challenging disease to treat. In the context of the increasing t-NEPC, ongoing efforts are needed to develop a better understanding of the genomic and epigenomic alterations that are associated with NEPC toward the development of new therapies.

## Figures and Tables

**Table 1:** Pertinent lab values/trends.

**Time from diagnosis (days)**	**0–26**	**47**	**62**	**71**	**78**	**82** ^a^	**96**	**120**	**122**
Total bilirubin (mg/dL)	0.6	0.8	1.1	1.5	—	1.9	0.9	9.4	8.2
AST (U/L)	33	38	64	118	—	179	34	322	485
ALT (U/L)	45	41	91	144	—	241	61	233	304
AlkPhos (U/L)	50	57	77	127	—	448	199	978	824
Creatinine (mg/dL)	0.7	0.9	0.79	0.75	—	0.75	0.66	3.98	5.19
PSA (ng/mL)	58.44	12.44	—	2.62^∗^	—	—	—	—	—
Chromogranin A (ng/mL)	—	—	—	—	32	—	—	—	—

Abbreviations: AlkPhos, alkaline phosphatase; ALT, alanine aminotransferase; AST, aspartate aminotransferase; PSA, prostate-specific antigen.

^a^Cycle 1: day 1 of carboplatin/etoposide therapy.

^*^Started leuprolide acetate on Day 57 (preceded by bicalutamide).

## Data Availability

No dataset was used for analysis. All relevant data/findings pertaining to this case are presented within the article.
